# The fecal microbiota of patients with primary biliary cholangitis (PBC) causes PBC-like liver lesions in mice and exacerbates liver damage in a mouse model of PBC

**DOI:** 10.1080/19490976.2024.2383353

**Published:** 2024-08-06

**Authors:** Huiyong Jiang, Ying Yu, Xiaoxiang Hu, Bingbing Du, Yini Shao, Feiyu Wang, Lifeng Chen, Ren Yan, Lanjuan Li, Longxian Lv

**Affiliations:** a State Key Laboratory for Diagnosis and Treatment of Infectious Diseases, National Clinical Research Center for Infectious Diseases, Collaborative Innovation Center for Diagnosis and Treatment of Infectious Diseases, The First Affiliated Hospital, College of Medicine, Zhejiang University, Hangzhou, China; b School of Public Health, Hangzhou Medical College, Hangzhou, China; c Microecological Laboratory, Jinan Microecological Biomedicine Shandong Laboratory, Jinan, China

**Keywords:** Primary biliary cholangitis, gut microbiota, fecal microbiota transplantation, metabolome, transcriptome

## Abstract

The role of the gut microbiota in the occurrence and progression of primary biliary cholangitis (PBC) is not fully understood. First, the fecal microbiota of patients with PBC (*n* = 4) (PBC-FMT) or healthy individuals (*n* = 3) (HC-FMT) was transplanted into pseudo germ-free mice or 2OA-BSA–induced PBC models. The functions, histology and transcriptome of the liver, and microbiota and metabolome of the feces were analyzed. Second, the liver transcriptomes of PBC patients (*n* = 7) and normal individuals (*n* = 7) were analyzed. Third, the liver transcriptomes of patients with other liver diseases were collected from online databases and compared with our human and mouse data. Our results showed that PBC-FMT increased the serum ALP concentration, total bile acid content, liver injury and number of disease-related pathways enriched with upregulated liver genes in pseudo germ-free mice and increased the serum glycylproline dipeptidyl aminopeptidase level and liver damage in a 2OA-BSA–induced PBC model. The gut microbiota and metabolome differed between PBC-FMT and HC-FMT mice and reflected those of their donors. PBC-FMT tended to upregulate hepatic immune and signal transduction pathways but downregulate metabolic pathways, as in some PBC patients. The hematopoietic cell lineage, Toll-like receptor, and PPAR signaling pathway were not affected in patients with alcoholic hepatitis, HBV, HCV, HCV cirrhosis, or NASH, indicating their potential roles in the gut microbiota affecting PBC. In conclusion, the altered gut microbiota of PBC patients plays an important role in the occurrence and progression of PBC. The improvement of the gut microbiota is worthy of in-depth research and promotion as a critical aspect of PBC prevention and treatment.

## Introduction

Primary biliary cholangitis (PBC) is an autoimmune liver disease characterized by progressive, nonsuppurative, and destructive intrahepatic cholangitis that mainly occurs in women.^1^ Its prevalence in different regions ranges from 1.91 to 40.2 per 100,000 inhabitants and shows an overall rapid upward trend.^2^ The etiology and pathogenesis of PBC are not fully understood. Currently, the main hypothesis is that PBC is caused by inappropriate immune responses (in genetically susceptible individuals) stimulated by environmental factors (microorganisms or xenobiotics), in which autoimmune immunity plays an important role.^3^ PBC cannot be cured at present, and two commonly used drugs, ursodeoxycholic acid (UDCA) and obeticholic acid, are both used for improving cholestasis.^4^ As a lifelong disease, if not well controlled, PBC progresses to cirrhosis and liver failure.^5^

Microorganisms are strongly associated with the destruction of immune tolerance and the pathogenesis of PBC.^6–8^ First, epidemiological studies have shown that a history of urinary tract infections (mainly *Escherichia coli* infection) is an important risk factor for PBC.^2^ Second, the molecular mimicry theory supports microorganisms as one trigger of PBC. The pyruvate dehydrogenase E2 component (PDC-E2) of *Escherichia coli* has significant homology with human PDC-E2.^9^ *Novosphingobium aromaticivorans* has two lipid-based proteins that are more homologous to human PDC-E2. Evidence of human PDC-E2–mimicking peptides and cross-reactive antibodies has also been observed in *Mycoplasma pneumoniae*, *Lactobacillus delbrueckii*, and *Mycobacterium gordonae*.^7^ Third, microbial byproducts can influence the development of PBC.^10^ For example, CpG in bacterial DNA, a natural ligand for TLR9, can promote autoimmunity by stimulating the innate immune system.^11^ These findings provide important insights into the pathogenesis and progression of PBC.

More than 70% of the human microbiota inhabits the gut and plays an important role in health and disease.^12^ We found that some potentially beneficial bacteria, such as *Ruminococcus bromii*, were absent in the gut of PBC patients, while some opportunistic pathogen-rich bacterial taxa, such as *Paraprevotella clara*, were enriched.^13^ Another study revealed that patients with PBC had a significantly reduced intestinal microbial diversity, and epithelial cell invasion pathways were strongly correlated with bacterial populations in *Enterobacteriaceae*.^14^ However, the role and mechanism of the gut microbiota in the occurrence and progression of PBC are not fully understood.

In this work, the effect of the gut microbiota of PBC patients on both healthy mice and a mouse model of PBC was explored by fecal microbiota transplantation (FMT); then, the potential mechanism by which the gut microbiota affects PBC was further investigated by comparing alterations in liver gene expression and pathways caused by FMT with those in PBC patients.

## Methods

**Figure 1.**
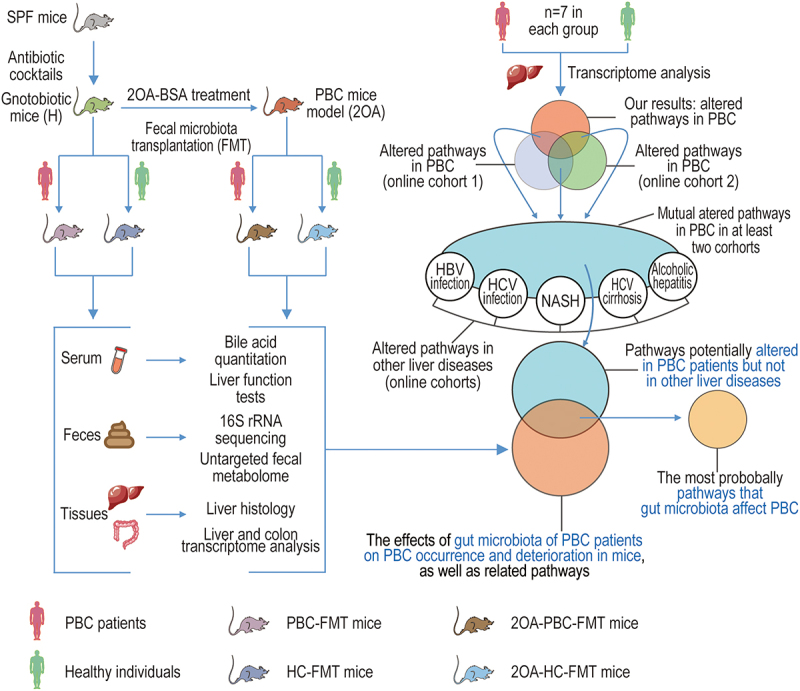
Experimental design scheme.

The overall experimental design scheme is shown in Figure 1.

### PBC patients and healthy controls

All enrolled PBC patients met the diagnostic guidelines of the American Association for the Study of Liver Diseases (AASLD) revised in 2018. The diagnosis of PBC is generally based on the presence of at least two of the following criteria: a. biochemical evidence of cholestasis with elevated ALP activity; b. presence of AMA; and c. histopathologic evidence of nonsuppurative cholangitis and destruction of small or medium-sized bile ducts if a biopsy is performed.^15^ Patients with any other disease, as well as those treated with biological agents, antibiotics, probiotics, immunosuppressants, or hormones, were excluded. The sex and age of the healthy controls were matched to those of the PBC group, and no antibiotics or probiotics were used within three months. Four PBC patients and three healthy controls were selected as continuous donors for FMT (each donor provided stool for FMT into 2–3 mice). Seven hepatic samples from PBC patients and seven normal hepatic samples from patients who underwent surgery for hemangioma were used for transcriptome studies (Tables S1 and S2). This study was approved by the Ethics Committee of the First Affiliated Hospital, Zhejiang University (IIT20210033A), and all participants signed informed consent forms.

### Animals

Six- to eight-week–old female C57BL/6 mice were purchased from Zhejiang Experimental Animal Center. Mice were fed standard chow in a temperature-controlled room (22 °C) with a humidity of 55% and a 12-hour light and dark cycle. The animal experiments were approved by the Ethics Committee of the First Affiliated Hospital of Zhejiang University (Zhejiang, China) and followed the NIH Guide for the Care and Use of Laboratory Animals. After the experiment, the mice were euthanized, and blood, colons, feces, and liver were collected. Except for those requiring immediate processing, the other samples were stored at −80°C until use.

### Fecal microbiota transplantation

Prior to FMT, C57BL/6 mice were treated with antibiotic cocktails for 27 days as described previously.^16^ In the first cycle, drinking water containing a mixture of nonabsorbable antibiotics (1 mg/mL each of ertapenem sodium, neomycin sulfate, and vancomycin hydrochloride) was administered for 7 days, and normal drinking water was administered for 2 days. In the second cycle, drinking water containing an absorbable antibiotic mixture (1 mg/mL each of ampicillin sodium, cefoperazone sodium, and clindamycin hydrochloride) was administered for 7 days, followed by 2 days of normal drinking water. In the third cycle, drinking water containing a mixture of nonabsorbable antibiotics was administered for another 7 days, and then normal drinking water was administered for 2 days.

After the third antibiotic cycle, the mice were gavaged with 100 μL of a microbial suspension once a week until the end of the experiment. Fresh feces from PBC patients or healthy controls were resuspended in normal saline that was preincubated in a 37°C anaerobic environment for 48 hours to prepare the microbial suspensions. Then, the mixture was centrifuged at 500 × g for 1 min to remove insoluble substances. The supernatant was filtered through a 70 μm filter. Afterward, the crude microbial suspension was washed twice using preincubated normal saline and adjusted to 1–3×10^9^ colony-forming units per milliliter using an HD-825-THOMA bacterial counting plate.^17^ The mice in the empty transplant group were transplanted only with normal saline.

### Establishment of the mouse model of PBC

**Figure 2.**
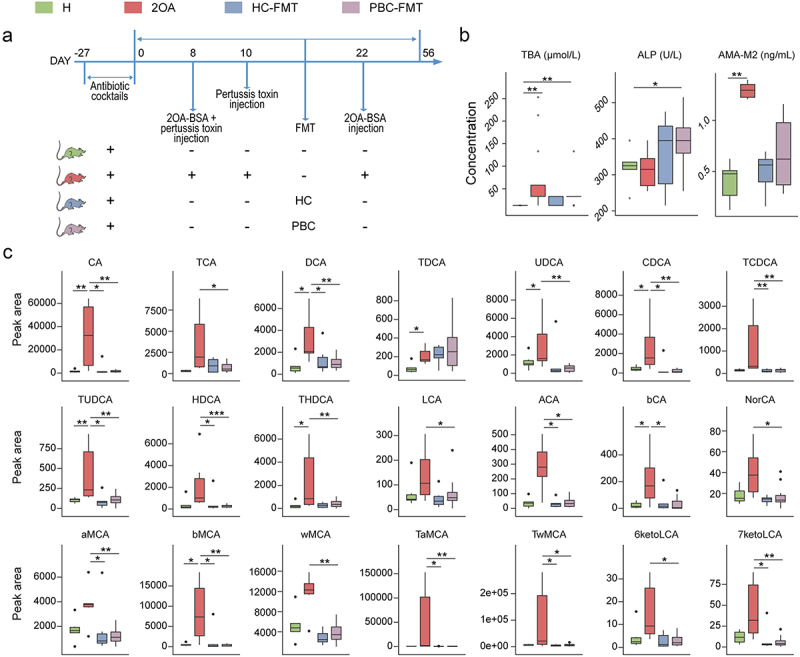
PBC-FMT increases the serum ALP and total bile acid levels in mice.

2-Octynoic acid-bovine serum albumin (2OA-BSA) was synthesized according to the literature.^18^ 2OA-BSA dissolved in Freund’s complete adjuvant was prepared by dissolving 100 μg of 2OA-BSA first in 50 μL of normal saline and then in 50 μL of Freund’s complete adjuvant. A similar method was used to prepare 2OA-BSA dissolved in Freund’s incomplete adjuvant. Female C57BL/6 mice were injected intraperitoneally with 100 μg of 2OA-BSA in Freund’s complete adjuvant, followed by an intraperitoneal injection of 100 ng of pertussis toxin to establish the mouse PBC model. After 14 days (the 15th day), 100 μg of 2OA-BSA in Freund’s incomplete adjuvant was injected intraperitoneally.^19^ Fecal and blood samples were collected at 0, 4, and 8 weeks, respectively; liver and colon samples were collected at 8 weeks (Figures 2a and 7a).

(a) Experimental design: Prior to FMT, C57BL/6 mice were treated with antibiotic cocktails for 27 days. Then, the mice were gavaged with a microbial suspension once a week for 8 weeks. 2OA-BSA and pertussis toxin were used to establish the PBC mouse model. (b) Concentrations of TBA, ALP and AMA-M2 in serum samples. (c) Quantitative analysis of 23 types of serum bile acids via UPLC‒MS/MS. *P < 0.05, **P < 0.01, and ***P < 0.001.

### Analyses of liver function and antimitochondrial antibody levels

Liver function indicators, such as serum levels of alanine aminotransferase (ALT), aspartate aminotransferase (AST), albumin, globulin, direct bilirubin, indirect bilirubin, γ-glutamyltransferase, glycylproline dipeptidyl aminopeptidase (GPDA) and other indicators, were determined using an automatic biochemical analyzer (7600–020, Hitachi Inc., Tokyo, Japan). Serum AMA-M2 levels were measured using a mouse AMA-M2 ELISA kit (ZCI BIO, Shanghai, China).

### Histological staining

Liver tissues were fixed with 10% neutral formaldehyde, dehydrated with different concentrations of ethanol, waxed, embedded in paraffin, cut into 4-μm thick sections, stained with hematoxylin and eosin (HE), and then scanned with a section scanner (PL250, 3D Histech). Then, portal vein inflammation, bile duct epithelial injury, nonpurulent destructive cholangitis, and bile duct loss were evaluated by 2 independent investigators in a blinded manner. Pathological scores (scale, 0–5) are presented as the means ± SEMs.

Immunohistochemical (IHC) staining was performed on paraffin-embedded liver tissue sections. The liver tissue sections were dewaxed, rehydrated, subjected to heat-induced antigen retrieval, and then subjected to blocking of endogenous peroxidase activity with 3% H_2_O_2_. Ten percent BSA was used to block the sections for 30 minutes. The sections were then incubated overnight at 4°C with primary antibodies (against CD4, 1:2000, Cat# ab183685, Abcam; CD8, 1:2000, Cat# ab209775, Abcam; CD11b, 1:40000, Cat# ab133357, Abcam; CD20, 1:100, Cat# ab64088, Abcam; and CD56, 1:500, Cat#99746, CST). The sections were washed and incubated with a secondary antibody (goat anti-rabbit IgG-HRP, 1:2000, Cat# ab205718, Abcam) at 37°C for 45 minutes. The sections were developed with DAB and then the nucleus was counterstained with hematoxylin. The immunohistochemical changes were photographed under an upright microscope.

### 16S rRNA gene sequencing and analysis

Fecal DNA was extracted from samples from patients, healthy controls, and mice using a PowerSoil DNA Isolation Kit (MO BIO Labs, CA, USA). The V3-V4 region of the 16S rRNA gene was amplified using the primer pair 338F (5’-ACTCCTACGGGAGGCAGCAG-3’) and 806 R (5’-GGACTACHVGGGTWTCTAAT-3’), whose 5’ ends were tagged with specific barcodes for each sample.^20^ The Illumina MiSeq platform (Illumina Inc., San Diego, CA) was used to sequence the resulting amplicons. Raw paired-end reads were assigned to the samples, which were merged, and poor-quality sequences were discarded. After the chimeric sequences were filtered, those with 97% identity were clustered into operational taxonomic units (OTUs) by Verseach v2.3.4. and assigned to each representative sequence using the RDP classifier (version 2.2) and Greengenes database (version 13.5). Diversity was analyzed using the QIIME 2 pipeline.

### Untargeted fecal metabolomics analysis using GC‒MS

The GC‒MS analysis was conducted as previously described.^21^ Briefly, 500 μL of chromatography-grade methanol prechilled at 4°C and 100 mg of ceramic beads (1 mm in diameter) were added to 50 mg of feces, and the mixture was homogenized three times using a Precellys Evolution homogenizer (Bertin Technologies, USA). Then, the mixtures were centrifuged at 18,000 × g for 15 min and filtered through a 0.22-μm membrane. Two hundred microliters of the supernatant was added to 20 μL of heptadecanoic acid (1 mg/mL) as an internal reference and dried under nitrogen at room temperature. Next, 50 μL of methoxy pyridine solution (15 mg/mL) was added to the dried mixture, which was then vortex mixed for 1 min, and stored air tight at 37°C for 24 h. Then, 50 μL of N,O-bis(trimethylsilyl)acetamide with 1% trimethylchlorosilane was added, and the mixture was thoroughly mixed and incubated at 70°C for 2 h for derivatization. The pretreated sample was analyzed with an Agilent 7890A-5975C GC‒MS system (Agilent, CA, USA).

### Quantitation of serum bile acid contents using UPLC-MS/MS

Twenty-three bile acid standards were obtained from Steraloids (Newport, RI, USA), TRC Chemicals (Toronto, ON, Canada), and Metabo-Profile (Shanghai, China). Ten isotope-labeled bile acid internal standards were purchased from C/D/N Isotopes (Quebec, Canada) and Steraloids (for abbreviations and full names of the 23 bile acids, see Table S3). An ultrahigh-performance liquid chromatography – tandem mass spectrometry (UPLC-MS/MS) system (ACQUITY UPLC-Xevo TQ-S, Waters Corp., Milford, MA, USA) was used to quantify bile acids in mouse blood samples, as described previously.^22^ The raw data exported from the UPLC‒MS/MS system were processed using QuanMET software (v1.0, Metabo-Profile, Shanghai, China). The actual concentration was obtained by comparing the quantitative curve of a bile acid in the sample with its standard.

### Transcriptome sequencing and analysis

RNA was extracted with TRIzol reagent (Invitrogen, USA). Libraries were constructed with the NEBNext Ultra RNA Library Prep Kit (NEB, USA). Both the building of the index of the reference genome and the alignment of paired-end clean reads to the reference genome were performed using HISAT2v2.0.5. StringTie (1.3.3b) was used for novel gene prediction. FeatureCounts (1.5.0-p3) was used to calculate the number of reads mapped to each gene. DESeq2 (1.16.1) was used for the analysis of differential expression between two comparison combinations. GO enrichment analysis and KEGG pathway enrichment analysis of differentially expressed genes were performed using clusterProfiler ba (3.4.4).

### Collection and consolidation analysis of online data

In addition to our own liver transcriptome sequencing data for PBC patients, we also obtained online liver transcriptome data for patients with other liver diseases and healthy controls. These online transcriptome data include those of patients with PBC (GSE79850),^23^ hepatitis B virus (HBV) infection (GSE83148),^24^ hepatitis C virus (HCV) infection (GSE15331),^25^ HCV cirrhosis (GSE6764),^26^ alcoholic hepatitis (GSE28619),^27^ and nonalcoholic steatohepatitis (NASH) (GSE164760),^28^ as well as one differentially expressed gene list for PBC patients.^29^

### Statistical analysis

The analysis was performed with IBM SPSS Statistics 25.0 software. The Shapiro‒Wilk test was performed to determine whether the data were normally distributed. If the data were normally distributed, one-way analysis of variance (ANOVA) was used for comparisons among multiple groups; otherwise, a nonparametric test was used for comparisons. The data are presented as the means ± SEMs. A two-tailed *P* < .05 was considered to indicate statistical significance.

## Results

### PBC-FMT increases serum ALP and total bile acid levels and liver injury in mice

After three months, the body weights of mice transplanted with feces from PBC patients (PBC-FMT) were not significantly different from those of mice transplanted with feces from healthy controls (HC-FMT). Liver function testing revealed a significant increase in the serum bile acid concentration in the 2OA-BSA–induced PBC mouse model. The serum AMA-M2 level was also significantly elevated in the 2OA-BSA–induced PBC mouse model (Figure 2(b)). PBC-FMT caused significant increases in the serum ALP and total bile acid levels, both of which are important indicators of PBC. In contrast, HC-FMT did not cause significant changes in liver function indicators in mice. Moreover, compared with those in HC-FMT mice, the serum bile acid levels in PBC-FMT mice tended to increase (*P* = .084). These results indicate that PBC-FMT, rather than HC-FMT, is prone to inducing increases in total bile acid and ALP levels in mice.

The quantitative UPLC-MS/MS analysis of serum bile acids revealed that the levels of many bile acids, such as cholic acid and chenodeoxycholic acid, were significantly increased in the 2OA-BSA treated mice compared with the healthy mice, suggesting the occurrence of cholestasis (Figure 2(c)). However, although we found that PBC-FMT caused an increase in total bile acid levels in mouse serum using the circulating enzyme method, as mentioned above, no significant increase was found in the 23 types of bile acids analyzed using UPLC-MS/MS. This discrepancy may be due to the differences in analysis methods and the limited types of bile acids we used. Overall, these findings indicate that the increase in total bile acid levels caused by PBC-FMT does not lead to cholestasis like the increase caused by 2OA-BSA.

**Figure 3.**
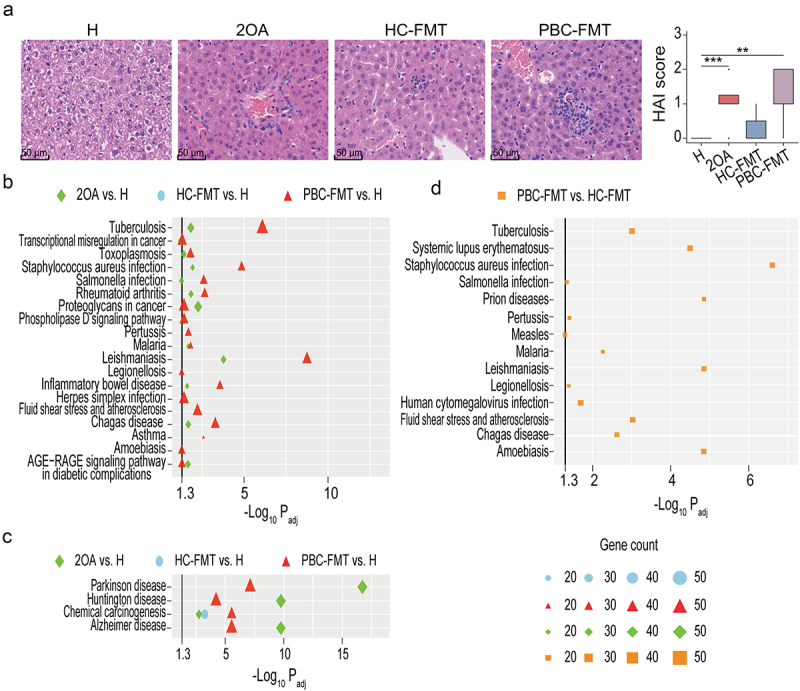
PBC-FMT increases liver injury in mice.

PBC-FMT caused liver lesions resembling cholangitis in mice. HE staining revealed that the livers of PBC-FMT mice exhibited inflammatory phenotypes, such as neutrophil and macrophage aggregation around the bile ducts and even a partial disappearance of small bile ducts (Figure 3(a)). This finding is very similar to the alterations in the small bile ducts of the 2OA-BSA–induced PBC mouse model. However, these liver lesions were not observed in HC-FMT mice, suggesting that PBC-FMT, rather than HC-FMT, can induce injury to small bile ducts in mice. Liver IHC staining revealed abundant CD4^+^ T cell, CD11b^+^ granulocyte and CD56^+^ NK cell infiltration around the bile ducts in the 2OA and PBC-FMT groups (Supplementary Figure S1).

(a) Representative images of hepatic H&E staining and histological scores of the livers; *P < 0.05, **P < 0.01, and ***P < 0.001. (b) Compared with those in healthy mice, the transcripts of genes whose expression in the liver was upregulated in 2OA-BSA–treated mice, HC-FMT mice or PBC-FMT mice were significantly enriched in many disease-related pathways. (c) Compared with those in healthy mice, the transcripts of genes whose expression decreased in 2OA-BSA–treated mice, HC-FMT mice or PBC-FMT mice were significantly enriched in several disease-related pathways. (d) Compared with those in HC-FMT mice, only the genes whose transcripts were upregulated in PBC-FMT mice were significantly enriched in some disease-related pathways; black vertical lines represent -log_10_*P*_*adj*_ = 1.3, corresponding to *P*_*adj*_ = 0.05.

Next, we further explored the different effects of PBC-FMT and HC-FMT on the liver by comparing disease-related pathways enriched in differentially expressed liver genes. Compared with those in healthy mice, the upregulated liver genes in 2OA-BSA–treated mice were significantly enriched in several immune disease pathways (inflammatory bowel disease and rheumatoid arthritis), infectious disease pathways (*Salmonella* infection, leishmaniasis, Chagas disease, malaria, toxoplasmosis, *Staphylococcus aureus* infection and tuberculosis), and cancer pathways (transcriptional misregulation in cancer, proteoglycans in cancer, and microRNAs in cancer) (Figure 3(b)); the downregulated genes were significantly enriched in 3 neurodegenerative diseases (Alzheimer’s disease, Parkinson’s disease, and Huntington’s disease) and the chemical carcinogenesis pathway (Figure 3(c)). Importantly, compared with those in healthy mice, the pathways enriched in liver genes whose transcripts were upregulated in PBC-FMT mice included nearly all pathways (except the phospholipase D signaling pathway and microRNAs in cancer) identified in 2OA-BSA–treated mice and included new pathways related to asthma, infectious diseases (pertussis, legionellosis, amoebiasis, and herpes simplex infection), fluid shear stress and atherosclerosis, and the AGE-RAGE signaling pathway in diabetic complications. Compared with those in 2OA-BSA–treated mice, the differentially expressed liver genes in PBC-FMT mice were not significantly enriched in any disease pathway. However, for all liver genes whose expression differed between HC-FMT mice and healthy mice, only genes whose expression decreased were significantly enriched in one pathway, chemical carcinogenesis (Figure 3(c)). Furthermore, compared with those in the HC-FMT mice, the disease pathways enriched in the liver genes whose transcripts were upregulated in the PBC-FMT mice included systemic lupus erythematosus, fluid shear stress and atherosclerosis, and infectious pathways (*Staphylococcus aureus* infection, tuberculosis, pertussis, legionellosis, *Salmonella* infection, leishmaniasis, amoebiasis, Chagas disease, malaria, human cytomegalovirus infection, measles, and prion diseases) (Figure 3(d)). These results clearly indicated that PBC-FMT–induced alterations in the mouse liver are similar to those induced by 2OA-BSA.

### FMT mice reflected differences in gut microbiota between PBC patients and HCs

**Figure 4.**
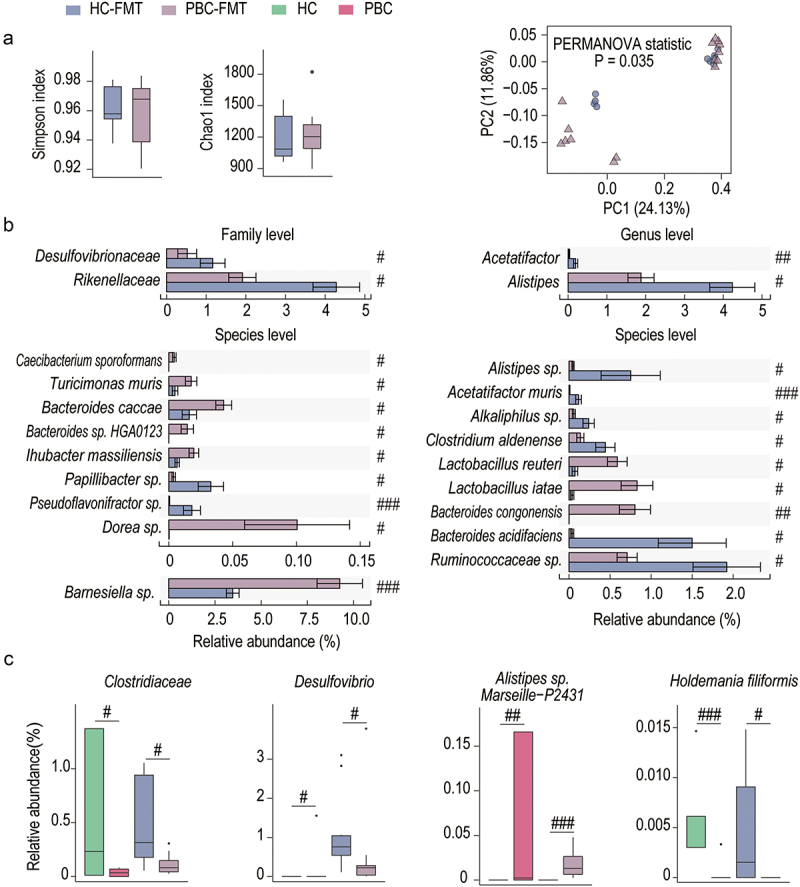
Differences in the gut microbiota between PBC patients and HCs are detected in FMT mice.

We first analyzed the fecal microbiota of mice after FMT to determine which microbes may play a role in PBC-FMT–related liver injury. According to the α diversity analysis, neither the Simpson nor Chao1 index differed between the PBC-FMT and HC-FMT mouse groups, indicating that the species evenness and richness of the two groups were similar (Figure 4(a)). In the PCoA, the spots of PBC-FMT and HC-FMT mice were significantly separated, indicating a significant difference in the microbiota structure between these two groups. This result was validated by permutational multivariate analysis of variance (PERMANOVA) (*P* =0 .035) (Figure 4(a)).

(a) Chao 1 index plot, Shannon index plot, and PCoA plot based on the gut microbiota operational taxonomic units (OTUs) of PBC-FMT mice and HC-FMT mice. (b) Families, genera and species that were differentially distributed between the PBC-FMT mice and the HC-FMT mice. (c) Differences in the gut microbiota between PBC patients and HCs, which were consistent with the differences in the gut microbiota between PBC-FMT and HC-FMT mice; # *P*_*adj*_ < 0.05, ## *P*_*adj*_ < 0.01 and ### *P*_*adj*_ < 0.001.

Compared with those in the gut of HC-FMT mice, some bacteria were highly enriched in the gut of PBC-FMT mice. For example, the species *Bacteroides* sp. HGA0123, *Alistipes* sp. *Marseille*–P2431 and *Caecibacterium sporoformans* were detected only in PBC-FMT mice. The relative abundances of *Bacteroides congonensis* and *Dorea* sp. in the gut of PBC-FMT mice were nearly 4014-fold and 503-fold higher than those in the gut of HC-FMT mice. The mean relative abundance of *Barnesiella* sp. increased from 3.46% in HC-FMT mice to 9.25% in PBC-FMT mice. In addition, *Bacteroides caccae*, *Lactobacillus reuteri*, *Lactobacillus iatae*, *Ihubacter massiliensis* and *Turicimonas muris* were also enriched in the gut of PBC-FMT mice. Moreover, compared with those in HC-FMT mice, some bacteria were depleted in the gut of PBC-FMT mice. Among them, *Holdemania filiformis* was found only in HC-FMT mice. The relative abundances of *Bacteroides acidifaciens*, *Acetatifactor muris* and *Pseudoflavonifractor* in PBC-FMT mice were only approximately 10% of those in HC-FMT mice. In addition, *Alkaliphilus* sp., *Clostridium aldenense*, *Papillibacter* sp. and *Holdemania filiformis* were also depleted in the gut of PBC-FMT mice (Figure 4(b)).

Next, we analyzed the differences in the gut microbiota between the feces from PBC patients and HCs used for gavage and found that they were partially consistent with the differences in the gut microbiota between PBC-FMT and HC-FMT mice. For example, *Clostridiaceae* tended to be depleted (*P* = .03, *P*_adj_ = 0.07); *Desulfovibrio* tended to be enriched (*P* = .02, *P*_adj_ = 0.08); members of *Bacteroidetes* such as *Alistipes* sp. Marseille P2431 were enriched; and *Holdemania filiformis* was depleted. However, some differences in the gut microbiota were also observed between PBC patients and HCs that were not detected in FMT-treated mice, such as the depletion of *Ruminococcus bromii* and enrichment of *Streptococcus constellatus* (Figure 4(c)).

### The gut metabolome of PBC-FMT mice differs from that of HC-FMT mice and is associated with altered microbes

**Figure 5.**
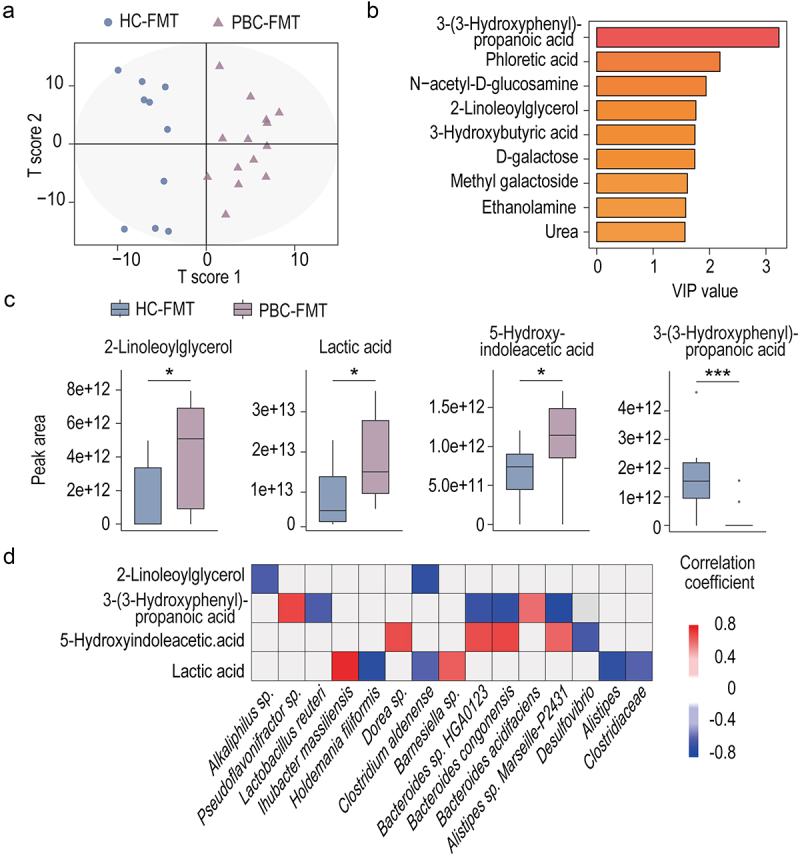
The gut metabolome of PBC-FMT mice differs from that of HC-FMT mice and is associated with altered microbes.

The gut microbiota and metabolism are closely linked, and thus differences in the fecal metabolome between PBC-FMT mice and HC-FMT mice were analyzed using GC‒MS. In total, 85 compounds were identified. OPLS-DA revealed that samples from PBC-FMT mice and HC-FMT mice were clearly separated, indicating significant differences in their metabolome profiles (Figure 5(a)). The variable importance in projection (VIP) value can be used to explain the contribution of variables to the model. Using a VIP greater than 1.5 as the threshold, metabolites such as 3-(3-hydroxyphenyl) propanoic acid, phloretic acid and N-acetyl-D-glucosamine were identified as important contributors (Figure 5(b)). Next, we compared the abundance of individual compounds in different groups. The results showed that lactic acid, 5-hydroxyindoleacetic acid, and 2-linoleoylglycerol were enriched in PBC-FMT mice, while 3-(3-hydroxyphenyl) propanoic acid was depleted (Figure 5(c)).

(a) OPLS-DA score plots of the metabolome profiles of PBC-FMT mice and HC-FMT mice. (b) Metabolites selected based on VIP values greater than 1.5. (c) Peak areas in the GC – MS analysis of significantly different metabolites between the PBC-FMT mice and HC-FMT mice; *P < 0.05, **P < 0.01, and ***P < 0.001. (d) Correlations of altered gut bacteria with altered gut metabolites (*P*_*adj*_ < 0.05).

We correlated the abovementioned metabolites with the differentially abundant gut microbes between PBC-FMT mice and HC-FMT mice and screened the results using *P* < 0.05 and an absolute correlation coefficient greater than 0.4 as thresholds. PBC-FMT–enriched *Bacteroides congonensis*, *Bacteroides* sp. HGA0123, and *Alistipes* sp. Marseille-P2431 were positively correlated with PBC-FMT–enriched 5-hydroxyindoleacetic acid and negatively correlated with PBC-FMT–depleted 3-(3-hydroxyphenyl) propanoic acid; moreover, *Dorea* sp. was positively correlated with 5-hydroxyindoleacetic acid, while *Lactobacillus reuteri* was negatively correlated with 3-(3-hydroxyphenyl) protonic acid. Furthermore, the abundances of PBC-FMT–enriched *Ihubacter massiliensis* and *Barnesiella* sp. were positively correlated with PBC-FMT–enriched lactic acid, while the abundances of PBC-FMT–depleted gut microbes were mainly negatively correlated with lactic acid. In addition, *Bacteroides acidifaciens* and *Pseudoflavonifractor* sp. were positively correlated with 3-(3-hydroxyphenyl) protonic acid, while *Alkaliphilus* sp. and *Clostridium aldenense* were negatively correlated with 2-linoleoylglycerol (Figure 5(d)).

### PBC-FMT tends to upregulate immune activity and signal transduction but decreases metabolism in the mouse liver

**Figure 6.**
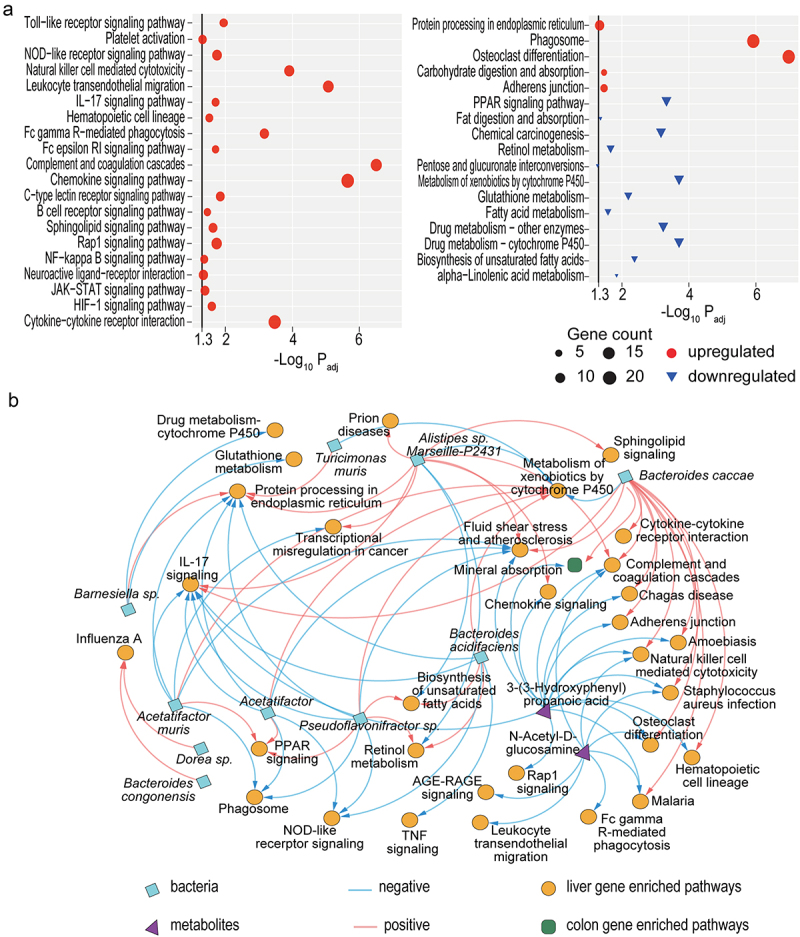
PBC-FMT tends to upregulate immunity and signal transduction but downregulates metabolism in the mouse liver.

Compared with those of healthy mice, the altered transcripts of liver genes in PBC-FMT mice, HC-FMT mice, or 2OA-treated mice were enriched in 75, 15, and 67 pathways, respectively (Supplementary Figure S2). Among these pathways, 53 pathways were activated in both the PBC-FMT group and the 2OA-treated group, indicating that these two treatments may involve several common mechanisms. In addition to genes involved in the abovementioned disease pathways, the altered transcripts for liver genes from PBC-FMT mice compared to HC-FMT mice were enriched mainly in pathways related to immune, metabolic, and signal transduction pathways (Figure 6(a)). First, the liver genes whose expression was upregulated in PBC-FMT mice were enriched in 13 immune pathways, such as natural killer cell-mediated cytotoxicity, hematopoietic cell lineage, leukocyte transendothelial migration, the Toll-like receptor signaling pathway and the IL-17 signaling pathway, as well as 7 signal transduction pathways, such as the JAK-STAT signaling pathway, the NF-kappa B signaling pathway and the cytokine‒cytokine receptor interaction pathway. Second, the downregulated liver genes in PBC-FMT mice were enriched in 9 metabolic pathways, such as drug metabolism-cytochrome P450, metabolism of xenobiotics by cytochrome P450 and retinol metabolism. Moreover, the genes whose expression was upregulated in the livers of PBC-FMT mice were also enriched in the terms “adherens junction”, “protein processing in endoplasmic reticulum” and “osteoclast differentiation”, while those whose expression was downregulated were also enriched in the PPAR signaling pathway and fat digestion and absorption pathway.

(a) Enriched immune, signal transduction and metabolic pathways related to the upregulated or downregulated liver genes from PBC-FMT mice compared to HC-FMT mice. The black vertical lines represent -log_10_*P*_*adj*_ = 1.3, corresponding to *P*_*adj*_ = 0.05. (b) Correlations of differentially distributed gut microbes and metabolites with differentially expressed liver or colon genes in enriched pathways between PBC-FMT and HC-FMT mice (*P*_*adj*_ < 0.05).

Next, the correlation of differentially distributed gut microbes and metabolites with differentially transcribed liver genes between PBC-FMT and HC-FMT mice was analyzed using the Spearman method. The liver genes that were positively or negatively correlated with certain microbes or metabolites were subsequently subjected to the Metascape tool to identify relevant pathways. First, positive correlations were mainly observed for liver genes mapped in diseases and immune pathways with PBC-FMT–enriched members of Bacteroidetes, such as *Bacteroides caccae* with *Staphylococcus aureus* infection, amoebiasis, malaria, Chagas disease, adherens junction, and osteoclast differentiation;* Alistipes* sp. Marseille-P2431 with prion diseases and transcriptional misregulation in cancer;* Bacteroides caccae* and *Alistipes sp*. Marseille-P2431 with fluid shear stress and atherosclerosis, natural killer cell-mediated cytotoxicity, complement and coagulation cascades, and the IL-17 signaling pathway; and *Bacteroides congonensis* and *Dorea* sp. with influenza A. Second, negative correlations were mainly observed for liver genes mapped in metabolic pathways with members of Bacteroidetes and for liver genes mapped in immune pathways with members of Firmicutes, such as *Bacteroides caccae*, *Barnesiella sp*., *Alistipes sp*. Marseille-P2431 and *Turicimonas muris* with xenobiotic biodegradation and metabolism; *Alistipes* sp. Marseille P2431 with biosynthesis of unsaturated fatty acids and retinol metabolism; and *Bacteroides acidifaciens*, *Acetatifactor muris*, and *Pseudoflavonifractor sp*. with the NOD-like receptor signaling pathway and IL-17 signaling pathway. Third, differentially abundant gut metabolites, such as 3-(3-hydroxyphenyl)propanoic acid, N-acetyl-D-glucosamine, which are involved in osteoclast differentiation, natural killer cell-mediated cytotoxicity, *Staphylococcus aureus* infection, complement and coagulation cascades, and malaria, were negatively correlated with liver genes associated with diseases and immune pathways; 3-(3-hydroxyphenyl)propanoic acid correlated with the chemokine signaling pathway, fluid shear stress and atherosclerosis, amoebiasis, IL-17 signaling pathway, Chagas disease, adherens junction, Rap1 signaling pathway, and hematopoietic cell lineage; N-Acetyl-D-glucosamine correlated with Fc gamma R-mediated phagocytosis, leukocyte transendothelial migration, and the AGE-RAGE signaling pathway in diabetic complications (Figure 6(b)).

### Colonic genes with upregulated expression in PBC-FMT mice were significantly enriched only in the mineral absorption pathway

Compared with those in healthy mice, the colonic genes whose transcripts differed among PBC-FMT–treated, HC-FMT–treated, and 2OA-treated mice were enriched in 86, 45 and 65 pathways, respectively (Supplementary Figure S3). Compared with those in HC-FMT mice, the genes whose transcripts were upregulated in the colon of PBC-FMT mice were significantly enriched only in the mineral absorption pathway, while the downregulated genes were not significantly enriched in any pathway. The colonic genes enriched in mineral absorption were positively correlated with *Bacteroides caccae* but negatively correlated with the gut 3-(3-hydroxyphenyl) propanoic acid level (Figure 6(b)).

### PBC-FMT exacerbates liver damage in a mouse model of PBC

**Figure 7.**
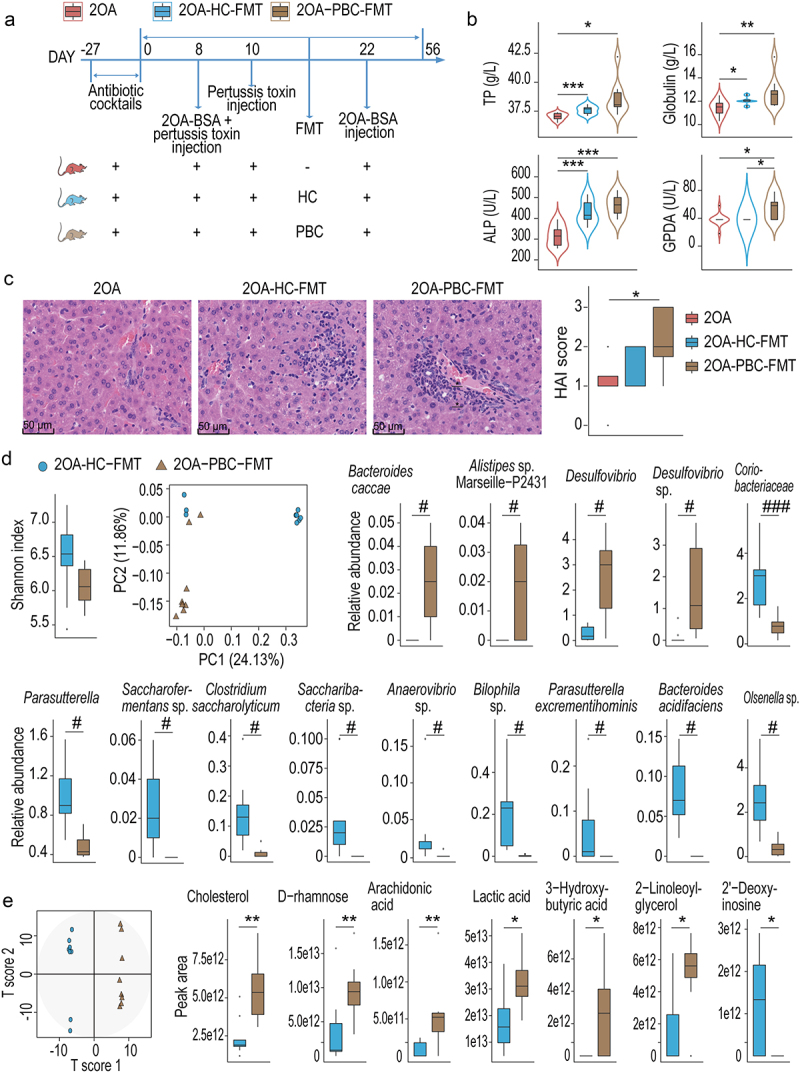
PBC-FMT exacerbates liver damage in a mouse model of PBC.

After PBC-FMT or HC-FMT, the levels of TP, globulin and ALP were increased in the 2OA-BSA–induced PBC model mice. Furthermore, compared with those in the 2OA-BSA–induced PBC mouse model (the 2OA group) and the corresponding HC-FMT group (the 2OA-HC–FMT group), the serum GPDA level in the PBC-FMT group (the 2OA-PBC–FMT group) was higher (Figure 7(b)). A pathological comparison revealed that compared with HC-FMT, PBC-FMT induced more aggregation of inflammatory cells in the mouse model of PBC (Figure 7(c)). IHC staining of the liver showed that the aggregated inflammatory cells in the liver of the 2OA-PBC–FMT group were mainly CD4^+^ T cells, CD11b^+^ granulocytes and CD56^+^ NK cells, with small amounts of CD8^+^ T cells and CD20^+^ B cells (Supplementary Figure S1).

(a) Experimental design: Prior to FMT, C57BL/6 mice were treated with antibiotic cocktails for 27 days. Then, the mice were gavaged with a microbial suspension once a week for 8 weeks. 2OA-BSA and pertussis toxin were used to establish the PBC mouse model. (b) Concentrations of TP, globulin, ALP and GDPA in serum samples. (c) Representative images of hepatic H&E staining and histological scores of the livers. (d) Shannon index, PCoA plot and alterations in the relative abundances of bacterial taxa between the 2OA-PBC–FMT and 2OA-HC–FMT mice. (e) OPLS-DA score plots of metabolome profiles and peak areas of metabolites that were significantly different between the 2OA-PBC–FMT and 2OA-HC–FMT mice; *P < 0.05, **P < 0.01, ***P < 0.001, # *P*_*adj*_ < 0.05, ## *P*_*adj*_ < 0.01 and ### *P*_*adj*_ < 0.001.

The gut microbiota of the 2OA-PBC–FMT group differed significantly from that of the 2OA-HC–FMT group. The Shannon index of the gut microbiota tended to decrease (*P* =0 .094) from the former to the latter. PCoA plots showed significant differences in the microbiota profiles between the two groups. *Bacteroides cacca*e and *Alistipes* sp. Marseille P2431, belonging to Bacteroidetes, were detected only in the gut of 2OA-PBC–FMT mice. The average relative abundances of *Desulfovibrio* and *Desulfovibrio* sp. LNB1 increased more than tenfold from the 2OA-HC–FMT group to the 2OA-PBC–FMT group. Moreover, *Bacteroide*s *acidifaciens*, *Clostridium saccharolyticum*, and *Parasutterella excrementihominis* were depleted in the 2OA-PBC–FMT group (Figure 7(d)).

OPLS-DA plots clearly distinguished the gut metabolome profiles of the 2OA-HC–FMT group from those of the 2OA-PBC–FMT group. Compared with those in the former group, cholesterol, D-(-)-rhamnose, arachidonic acid, lactic acid, 3-hydroxybutyric acid, and 2-linoleoylglycerol were enriched, while 2’-deoxyinosine was depleted in mice from the 2OA-PBC–FMT group (Figure 7(e)).

Liver genes whose transcription was altered after PBC-FMT into mice in the PBC model group were enriched in more pathways than those whose expression was altered after HC-FMT. Compared with those in the 2OA group, liver genes that were upregulated in the 2OA-HC–FMT group were enriched in five pathways involved in metabolism, such as lipid and fatty acid metabolism, while downregulated liver genes were enriched in three pathways, namely, the PPAR signaling pathway, retinol metabolism, and fatty acid degradation. However, liver genes that were upregulated in the 2OA-PBC–FMT group were enriched in 61 pathways, including 25 disease pathways, such as autoimmune thyroid disease, rheumatoid arthritis, asthma, systemic lupus erythematosus, and primary immunodeficiency; 16 immune system pathways, such as antigen processing and presentation and Th17 cell differentiation; and pathways such as NF-kappa B, TNF, JAK-STAT, p53, cell cycle, apoptosis and necroptosis. The genes whose expression decreased in the liver of the 2OA-PBC–FMT group were enriched in 10 pathways, 9 of which are metabolic pathways such as xenobiotic biodegradation and metabolism (Supplementary Figure S4).

Differentially expressed liver genes between the 2OA-PBC–FMT group and the 2OA-HC–FMT group were enriched in many disease, immune and metabolic pathways. In mice in the 2OA-PBC–FMT group, 33 pathways, including 16 disease pathways such as rheumatoid arthritis, inflammatory bowel disease, and systemic lupus erythematosus; 11 immune pathways such as hematopoietic cell lineage, the NOD-like receptor signaling pathway, and natural killer cell-mediated cytotoxicity; and several other pathways, such as cytokine‒cytokine receptor interaction and the NF-kappa B signaling pathway, were enriched in the upregulated liver genes. Moreover, the downregulated liver genes were enriched in 23 pathways, including 19 metabolic pathways related to lipids, carbohydrates, and xenobiotics, as well as several other pathways, such as bile secretion and cholesterol metabolism (Supplementary Figure S4).

### PBC-FMT–induced alterations in certain hepatic signaling pathways in mice are the same as those altered in PBC patients

Some pathways enriched in differentially expressed liver genes between PBC patients and HCs were significantly different from those enriched in patients with other liver diseases for which the transcriptome is available. We examined the liver transcriptomes of seven PBC patients and seven HCs who underwent surgery for hemangioma and compared our results with those from patients with other liver diseases. First, among the three liver transcriptome studies of PBC patients and HCs, including our study, 12 pathways enriched in genes upregulated by PBC were identified in at least two studies. These pathways included the hematopoietic cell lineage, primary immunodeficiency, the T-cell receptor signaling pathway, the PI3K-Akt signaling pathway, focal adhesion, leukocyte transendothelial migration, cytokine‒cytokine receptor interaction, graft-versus–host disease, natural killer cell-mediated cytotoxicity, the cell cycle, malaria, and the Toll-like receptor signaling pathway. Seven pathways enriched in downregulated liver genes in at least two studies were cytokine‒cytokine receptor interaction, the AGE-RAGE signaling pathway in diabetic complications, the prolactin signaling pathway, one carbon pool by folate, the PPAR signaling pathway, vitamin digestion and absorption, and acute myeloid leukemia. Second, the abovementioned PBC pathways had only 5 pathways identical to pathways enriched in the differentially transcribed liver genes from patients with HCV cirrhosis or patients with chronic hepatitis B compared with their HCs and had 2 pathways identical to those of patients with ALD or patients with NASH. These results indicate a specific mechanism of PBC occurrence and development (Table 1).

**Table 1.** Compared with those in healthy controls, the *P* values of pathways significantly enriched in the livers of patients or mice with different liver diseases.

Pathway	PBC patients^a^	PBC patients (response to UDCA) ^b^	PBC patients (nonresponse to UDCA) ^b^	PBC patients^c^	PBC-FMT mice^c^	2OA-PBC–FMT mice^c^	HCV- induced cirrhosis patients^d^	alcoholic hepatitis patients^e^	chronic hepatitis B patients^f^	nonalcoholic steatohepatitispatients^g^
Hematopoietic cell lineage	–	3.57E–28	1.72E–53	1.44E–03	3.90E–03	1.07E–08	–	–	–	–
Primary immunodeficiency	6.01E–04	8.00E–17	1.91E–23	2.76E–02	–	–	1.60E–08	–	–	–
T-cell receptor signaling pathway	–	–	3.72E–22	2.36E–03	2.82E–02	–	1.36E–06	–	–	–
PI3K-Akt signaling pathway	–	5.07E–07	2.60E–17	3.12E–02	–	–	–	–	–	–
Focal adhesion	–	–	1.24E–08	2.13E–05	–	–	–	–	–	–
Leukocyte transendothelial migration	–	–	1.21E–05	3.88E–02	2.42E–07	9.47E–05	2.20E–09	4.28E–08	4.53E–08	–
Cytokine‒cytokine receptor interaction	3.05E–08	1.33E–24	2.10E–40	–	1.88E–05	1.71E–03	–	–	3.69E–12	3.45E–03
Graft-versus–host disease	3.23E–09	–	–	4.95E–08	–	2.68E–07	–	–	–	–
Natural killer cell mediated cytotoxicity	8.65E–07	2.13E–08	4.09E–23	–	6.50E–06	1.99E–07	1.89E–08	–	7.10E–09	–
Cell cycle	1.08E–04	–	–	7.95E–05	–	–	–	4.44E–08	4.44E–08	–
Malaria	2.86E–04	2.27E–08	1.04E–06	–	4.31E–04	–	6.95E–03	–	1.44E–10	–
Toll-like receptor signaling pathway	2.97E–03	3.44E–09	–	–	9.70E–04	3.44E–03	–	–	–	–
Cytokine‒cytokine receptor interaction	6.67E–10	4.63E–32	1.60E–19	–	–	–	–	–	–	–
AGE-RAGE signaling pathway in diabetic complications	9.29E–09	8.91E–13	–	–	–	–	–	–	–	1.15E–05
Prolactin signaling pathway	2.72E–04	7.48E–05	–	–	–	–	–	–	–	–
One carbon pool by folate	2.19E–02	–	–	3.05E–03	–	–	–	–	–	–
PPAR signaling pathway	2.70E–02	–	–	2.14E–03	8.88E–06	–	–	–	–	–
Vitamin digestion and absorption	3.56E–02	–	–	3.24E–03	–	–	–	–	–	–
Acute myeloid leukemia	9.73E–04	–	1.72E–05	–	–	–	–	–	–	–

^a^Ueno K, Aiba Y, Hitomi Y, et al. Integrated GWAS and mRNA Microarray Analysis Identified IFNG and CD40L as the Central Upstream Regulators in Primary Biliary Cholangitis. Hepatol Commun 2020; 4(5): 724–38.

^b^Hardie C, Green K, Jopson L, et al. Early Molecular Stratification of High-risk Primary Biliary Cholangitis. EBioMedicine 2016; 14: 65–73.

^c^The data of this article.

^d^Wurmbach E, Chen YB, Khitrov G, et al. Genome-wide molecular profiles of HCV-induced dysplasia and hepatocellular carcinoma. Hepatology 2007; 45(4): 938–47.

^e^Affo S, Dominguez M, Lozano JJ, et al. Transcriptome analysis identifies TNF superfamily receptors as potential therapeutic targets in alcoholic hepatitis. Gut 2013; 62(3): 452–60.

^f^Zhou W, Ma Y, Zhang J, et al. Predictive model for inflammation grades of chronic hepatitis B: Large-scale analysis of clinical parameters and gene expressions. Liver Int 2017; 37(11): 1632–41.

^g^Lopez-Vicario C, Gonzalez-Periz A, Rius B, et al. Molecular interplay between Delta5/Delta6 desaturases and long-chain fatty acids in the pathogenesis of nonalcoholic steatohepatitis. Gut 2014; 63(2): 344–55.

PBC-FMT–induced alterations in certain hepatic signaling pathways in mice are similar to those in PBC patients, as mentioned above. Among the pathways enriched in differentially expressed liver genes between PBC patients and HCs, hematopoietic cell lineage, leukocyte transendothelial migration, cytokine‒cytokine receptor interaction, natural killer cell-mediated cytotoxicity, the Toll-like receptor signaling pathway, and PPAR signaling pathway were also observed in the comparisons of PBC-FMT mice with both HC-FMT mice and 2OA-PBC–FMT mice with 2OA-HC–FMT mice; the T-cell receptor signaling pathway and malaria were also found in the comparison of PBC-FMT mice and HC-FMT mice; and the graft-versus–host disease pathway was also found in the comparison of 2OA-PBC–FMT mice with 2OA-HC–FMT mice. In particular, the hematopoietic cell lineage, Toll-like receptor signaling pathway, graft-versus–host disease, and PPAR signaling pathway were not significantly enriched in pathways related to differentially transcribed liver genes between patients with ALD, HBV, HCV, HCV cirrhosis, NASH and their HCs, indicating that these four pathways may be involved in the mechanism by which the gut microbiota affects PBC (Table 1).

## Discussion

The role of the gut microbiota in the occurrence of PBC, as well as the long-term impact of a dysbiotic gut microbiota in PBC patients on health, are still unclear. This study transplanted feces from HC or PBC individuals into mice and found that the gut microbiota of mice after FMT reflected key differentially abundant bacteria between human fecal donors. Compared with HC-FMT mice, PBC-FMT mice had higher serum ALP and total bile acid levels, more severe liver injury, and significantly different gut metabolomes. According to the liver transcriptome analysis, PBC-FMT–upregulated liver genes were enriched in immune and signal transduction pathways, while downregulated genes were enriched in metabolic pathways. These characteristics were very similar to those of the 2-OA-induced PBC mouse model. Compared with HC-FMT–treated PBC model mice, PBC-FMT–treated PBC model mice exhibited increased serum GPDA levels, worsened liver inflammatory cell aggregation, upregulated liver genes mapped to disease and immune pathways, and downregulated liver genes mapped to metabolic pathways. Some PBC-FMT–related hepatic signaling pathways in mice, such as the hematopoietic cell lineage pathway, Toll-like receptor signaling pathway, graft-versus–host disease pathway, and PPAR signaling pathway, are the same as those altered in PBC patients but were not altered patients with ALD, HBV, HCV, HCV cirrhosis, or NASH for whom the transcriptome is available. These results preliminarily reveal the potential role and mechanism of the gut microbiota in the occurrence and development of PBC.

We found that the differences in the gut microbiota of mice after FMT partially reflected the differences in the gut microbiota between PBC patients and HCs. In most cases, the dysbiotic gut microbiota in patients is known to undergo transformation after transplantation to mice.^30^ This transformation is mainly due to the genetic, behavioral, physiological, and anatomical differences between humans and mice. We administered FMT once a week to minimize these effects and found that the gut microbiota of FMT mice reflects important features of the gut microbiota of PBC patients, such as the enrichment of members of Bacteroidetes. Some of these featured microbes have been shown to promote inflammation in other studies. For example, the genus *Alistipes*, to which *Alistipes sp*. Marseille-P2431 belongs, has the highest number of putrefaction pathways, typically leading to the bacterial production of harmful metabolites among commensal bacteria.^31^ Some members of *Alistipes*, such as *Alistipes putredinis* (CCUG 45,780 T) and *Alistipes finegoldii* (CCUG 46,020 T), are sufficient to induce colitis and site-specific tumors in IL10^–/–^ mice via the IL-6/STAT3 pathway.^32^ For another example, using a mouse model of spontaneous autoimmune myocarditis, Gil-Cruz et al. showed that the progression of myocarditis to lethal heart disease depends on cardiac myosin-specific TH17 cells imprinted in the intestine by a peptide mimic of the commensal *Bacteroides* species.^33^ Therefore, these results indicate that the mouse flora obtained after FMT has a material basis that reflects the pathological function of the microbiota of PBC patients.

Our results showed that compared to HC-FMT mice, PBC-FMT mice had higher levels of serum ALP and total bile acids; PBC-FMT induced higher levels of GPDA in PBC model mice than did HC-FMT. First, an increase in the serum total bile acid concentration suggested that PBC-FMT may cause cholestasis or local biliary obstruction. However, compared with 2-OA-BSA, PBC-FMT caused only a slight increase in bile acid levels and induced severe cholestasis in a mouse model of PBC. Second, for cholestatic patients, elevated ALP activity in the serum is an important diagnostic indicator of PBC. Moreover, if AMAs, other disease-specific autoantibodies, or histologic evidence is detected, patients can be diagnosed with PBC.^15^ Serum ALP mainly originates from the liver or bone. Any form of biliary obstruction can induce the synthesis of ALP by liver cells, leading to canalicular leakage of ALP into the hepatic sinusoid, which then flows into the blood vessels, causing an increase in serum ALP levels. Therefore, the increase in serum ALP levels caused by PBC-FMT may lead to hepatic/biliary injury.^34^ Third, serum GPDA activity increases in patients with liver diseases such as acute hepatitis, chronic viral hepatitis, cirrhosis, or obstructive jaundice. Moreover, the GPDA level has been shown to be elevated in the serum of patients with hepatocellular carcinoma and is proposed as a biomarker for diagnosing non-AFP–producing patients.^35^ Therefore, these alterations in serum indicators caused by PBC-FMT reflect the potential role of the gut microbiota in PBC, especially in bile duct injury.

Regulating metabolism is an important mechanism by which the gut microbiota affects health. Our results showed that PBC-FMT led to metabolic disorders in the gut of both mice and a mouse model of PBC. First, 5-hydroxyindoleacetic acid (5-HIAA), which was enriched in the feces of PBC-FMT mice, is the main metabolite of serotonin (from tryptophan). Fecal 5-HIAA is a key metabolic marker for intra-abdominal hypertension and gut-derived sepsis.^36^ Second, cholesterol, which was enriched in the feces of PBC-FMT mice, is crucial for the physiological functions of the immune and nervous systems. Similarly, liver genes downregulated by PBC-FMT were significantly enriched in the cholesterol metabolism pathway. Furthermore, the PPAR signaling pathway, which was enriched with downregulated genes in the livers of PBC-FMT mice, is an important factor regulating lipid metabolism, adipogenesis, insulin sensitivity, the inflammatory response, cell growth, and differentiation. It is an important target for PBC treatment, once again reflecting the role of the gut microbiota in regulating PBC through metabolism.^37^ Third, 3-(3-hydroxyphenyl) propanoic acid, which was depleted in PBC-FMT mice, is the main product of the conversion of phenols by the gut microbiota. It can be further decarboxylated by bacteria in the human gut or absorbed by the intestine and has various beneficial functions, such as antioxidant, anti-inflammatory, and bone resorption-inhibiting effects.^38^ Similarly, strong negative correlations between 3-(3-hydroxyphenyl) propanoic acid levels and altered liver genes involved in pathways such as the hematopoietic cell lineage, osteoclast differentiation, and the IL-17 signaling pathway were detected. Therefore, although current metabolic methods cannot reveal all altered metabolites, alterations in some metabolites with known functions have reflected the potential harm of an altered gut microbiota to PBC patients.

The enrichment of the upregulated liver genes in some immune pathways in both PBC patients and PBC-FMT mice indicates the potential mechanism by which the gut microbiota affects PBC. First, the involvement of the cytokine‒cytokine receptor interaction pathway reflects the close relationship between chronic liver inflammation and the gut microbiota.^1^ Second, the observed hematopoietic cell lineage pathway is involved in the differentiation of T cells, NK cells, basophils, macrophages and B cells. These cells play key roles in the occurrence and development of PBC.^39^ This finding indicates that regulating the hematopoietic cell lineage pathway is a potential working mechanism of the gut microbiota in PBC. Third, the leukocyte transdermal migration pathway is crucial for innate immunity and inflammation. When tissues are injured or infected, white blood cells can leave blood vessels by adhering to and detecting vascular endothelial cells, destroying endothelial cell – cell junctions, and migrating across the endothelium, initiating an inflammatory immune response.^40^ Therefore, the involvement of this pathway may at least explain how the gut microbiota exacerbates PBC. Fourth, the natural killer (NK) cell-mediated cytotoxicity pathway is an important effector mechanism of the immune system that combats viral infections and cancer. The frequency and cytotoxicity of NK cells increase in the livers of PBC patients. They accumulate around small bile ducts and induce apoptosis of biliary epithelial cells, thus promoting disease progression.^5,41^ Consequently, the gut microbiota may promote PBC through the NK cell-mediated cytotoxicity pathway. Finally, the Toll-like receptor signaling pathway plays an important role in the production of cytokines in autoimmune diseases, triggering or enhancing autoimmune processes, and is the core of cholestatic liver injury.^42^ It may also act as a key mechanism of PBC development. Therefore, the dysbiotic gut microbiota of PBC patients may affect PBC through these pathways, although further research on the detailed underlying mechanisms is needed.

In summary, the altered gut microbiota in PBC patients may play an important role in the occurrence and progression of PBC by regulating immune metabolism through pathways such as the hematopoietic cell lineage and the PPAR signaling pathway. The improvement of the gut microbiota is worthy of in-depth research and promotion as an important aspect of PBC prevention and treatment.

## Supplementary Material

Supplemental Material

## Data Availability

The sequence data were deposited into the NCBI Sequence Read Archive (SRA) (BioProject ID: PRJNA1055302) and the National Genomics Data Center of China (BioProject ID: PRJCA019524). The other datasets analyzed during the current study are available from the corresponding author upon reasonable request.
