# Supplementary material for: The fecal microbiota of patients with primary biliary cholangitis (PBC) causes PBC-like liver lesions in mice and exacerbates liver damage in a mouse model of PBC
Source: Gut Microbes. 2024 Aug 6;16(1):2383353. doi: 10.1080/19490976.2024.2383353 (PMC11305030; doi:10.1080/19490976.2024.2383353)
Supplement: Supplemental Material [file KGMI_A_2383353_SM2123.zip › Supplementary Materials.docx]

**Supplementary Table 1. Donor metadata. Metadata for seven donors whose samples were used for FMT.**

| **Donor ID** | **Group** | **Sex** | **Age**  **(years)** | **ALT**  **(U/L)** | **AST**  **(U/L)** | **ALP**  **(U/L)** | **TB**  **(μmol/L)** | **DB**  **(μmol/L)** | **GGT**  **(U/L)** |  |
| --- | --- | --- | --- | --- | --- | --- | --- | --- | --- | --- |
| HC-1 | | FMT-HC | F | 52 | 15 | 18 | 63 | 9 | 3 | 15 |
| HC-2 | | FMT-HC | F | 53 | 12 | 12 | 64 | 5 | 2 | 12 |
| HC-3 | | FMT-HC | F | 50 | 14 | 20 | 71 | 7 | 3 | 13 |
| PBC-1 | | FMT-PBC | F | 40 | 20 | 21 | 95 | 9 | 5 | 32 |
| PBC-2 | | FMT-PBC | F | 54 | 24 | 25 | 109 | 10 | 5 | 134 |
| PBC-3 | | FMT-PBC | M | 63 | 38 | 46 | 210 | 16 | 8 | 247 |
| PBC-4 | | FMT-PBC | F | 58 | 29 | 27 | 135 | 9 | 3 | 169 |

**Supplementary Table 2. Comparison of clinical features between PBC patients and healthy controls used for transcriptome studies.**

|  | **PBC patients**  **(n=7)** | **Healthy controls**  **(n=7)** | ***P* Value** |
| --- | --- | --- | --- |
| Median age (years) | 54 (38, 73) | 48 (41, 56) | 1.62E-01 |
| Alkaline phosphatase (U/L) | 186 (62, 389) | 71 (53, 129) | 3.30E-02 |
| Cholinesterase(U/L) | 1568 (970, 2445) | 6879 (2671, 9354) | 1.17E-03 |
| Total bile acid (μmol/L) | 304.2 (93.1, 590) | 3.9 (1.8, 6.4) | 2.09E-03 |
| Total bilirubin (μmol/L) | 220.3 (58.9, 531.7) | 9.3 (3, 16.8) | 9.03E-03 |
| Direct bilirubin (μmol/L) | 170.4 (47, 468) | 3.8 (1.5, 7.7) | 9.80E-03 |
| Adenylate deaminase (U/L) | 23 (14.6, 28.1) | 8.7 (7.5, 16.3) | 5.18E-05 |
| Total cholesterol (mmol/L) | 1.73 (0.86, 2.70) | 4.01 (2.47, 6.71) | 3.76E-03 |
| High-density lipoprotein (mmol/L) | 0.18 (0.08, 0.38) | 1.03 (0.61, 1.98) | 1.61E-03 |
| Low-density lipoprotein (mmol/L) | 0.65 (0.10, 1.33) | 2.11 (1.22, 3.81) | 5.63E-03 |
| α-L-fucosidase (U/L) | 35.8 (18.9, 57.2) | 19.4 (13.5, 23.5) | 2.11E-02 |
| Thrombin time(s) | 21.1 (17.7, 24.7) | 16.7 (16.0, 18.1) | 1.93E-03 |
| Prothrombin time(s) | 15.8 (13.1, 21.0) | 10.9 (10.2, 12.4) | 3.36E-03 |
| Activated partial thromboplastin time(s) | 50.4 (29.0, 75.8) | 26.1 (23.1, 29.6) | 1.14E-02 |

**Supplementary Table 3. Abbreviations and full names of 23 bile acids.**

| Abbreviation | Definition |
| --- | --- |
| TDCA | taurodeoxycholate |
| CA | cholic acid |
| LCA | lithocholic acid |
| HDCA | α-hyodeoxycholic acid |
| CDCA | chenodeoxycholic acid |
| TCA | taurocholic acid |
| TCDCA | taurochenodeoxycholate |
| UDCA_M | ursodeoxycholic acid |
| muroCA | murocholic acid |
| aMCA_M | α-muricholic acid |
| bMCA_M | β-muricholic acid |
| wMCA | ω-muricholic acid |
| TUDCA_M | tauroursodeoxycholic acid |
| THDCA | taurohyodeoxycholic acid |
| TaMCA_M | tauro α-muricholate |
| TbMCA_M | tauro β-muricholate |
| TwMCA | tauro ω-muricholate |
| NorCA | norcholic acid |
| DCA | deoxycholic acid |
| bCA | 3β-cholic acid |
| ACA | allocholic acid |
| 6_ketoLCA | 6-ketolithocholic acid |
| 7_ketoLCA | 7-ketolithocholic acid |


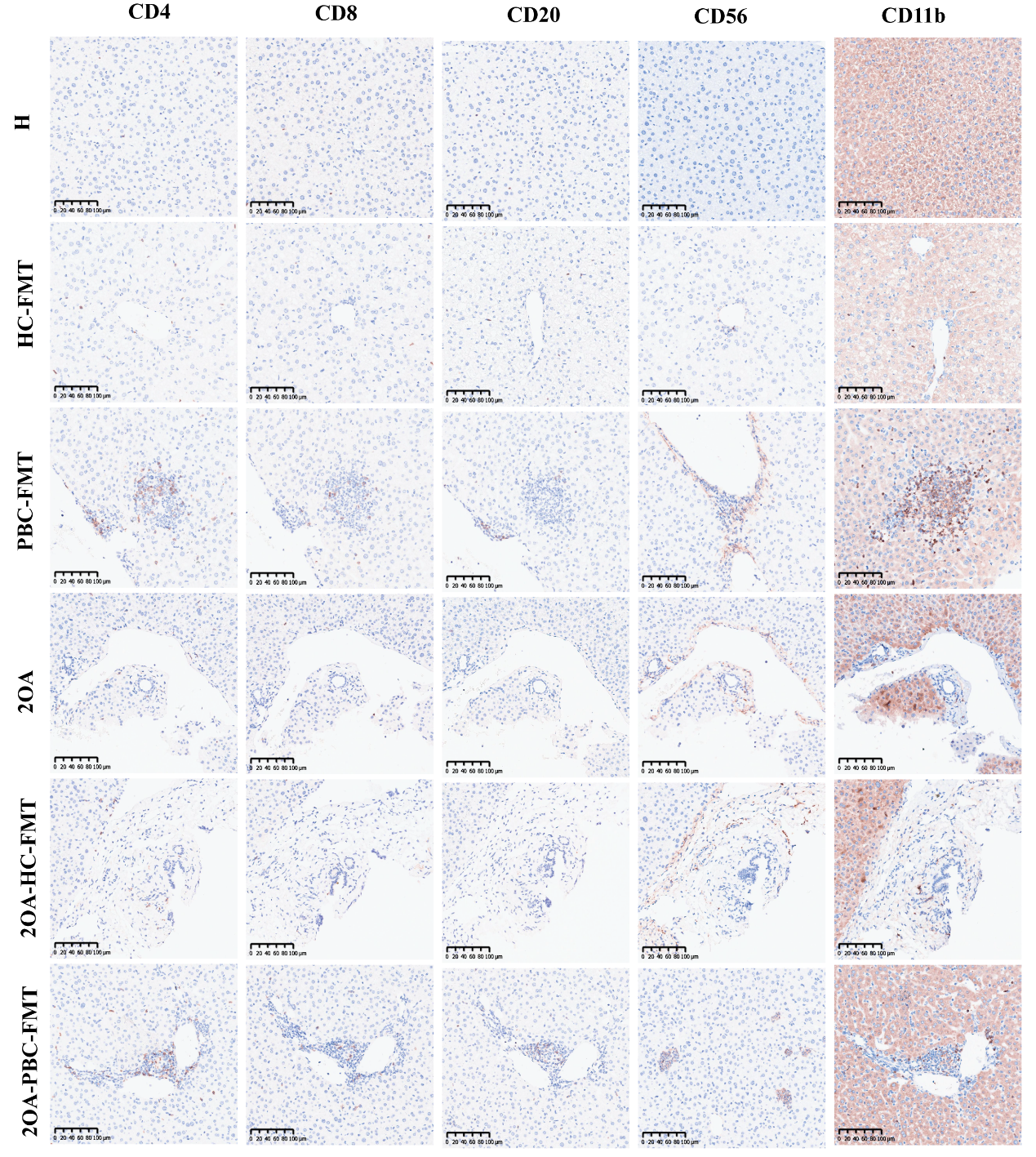


**Supplementary Figure 1. Immunohistochemical staining for CD4, CD8, CD20, CD56 and CD11b in the livers of the H, HC-FMT, PBC-FMT, 2OA, 2OA-HC-FMT and 2OA-PBC-FMT groups. Magnification: ×20, scale bar: 100 μm.**


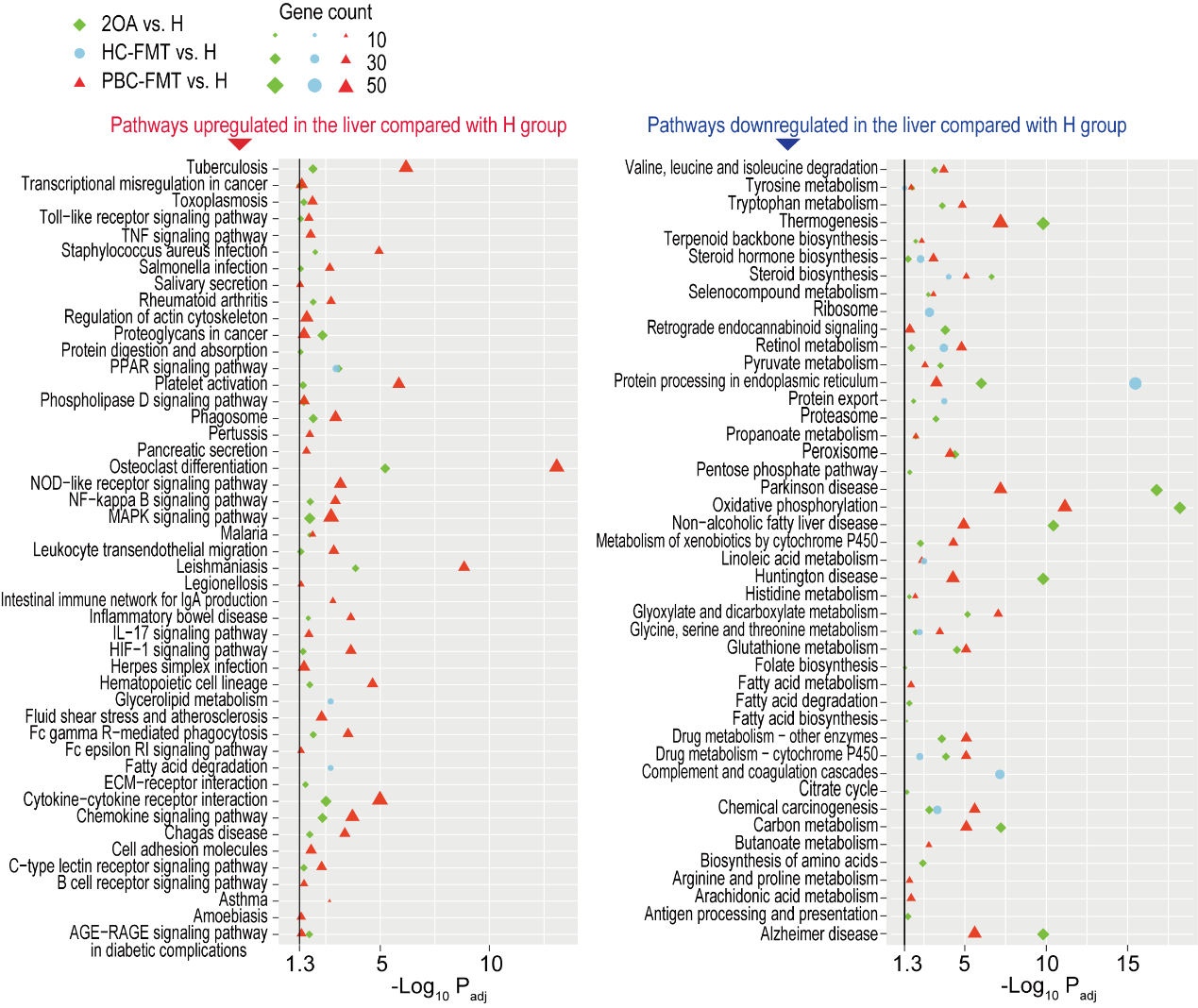


**Supplementary Figure 2. Compared with those of healthy mice, the altered transcripts for liver genes in PBC-FMT-treated mice, HC-FMT-treated mice, or 2OA-treated mice were enriched in 75, 15, and 67 pathways, respectively.**

**
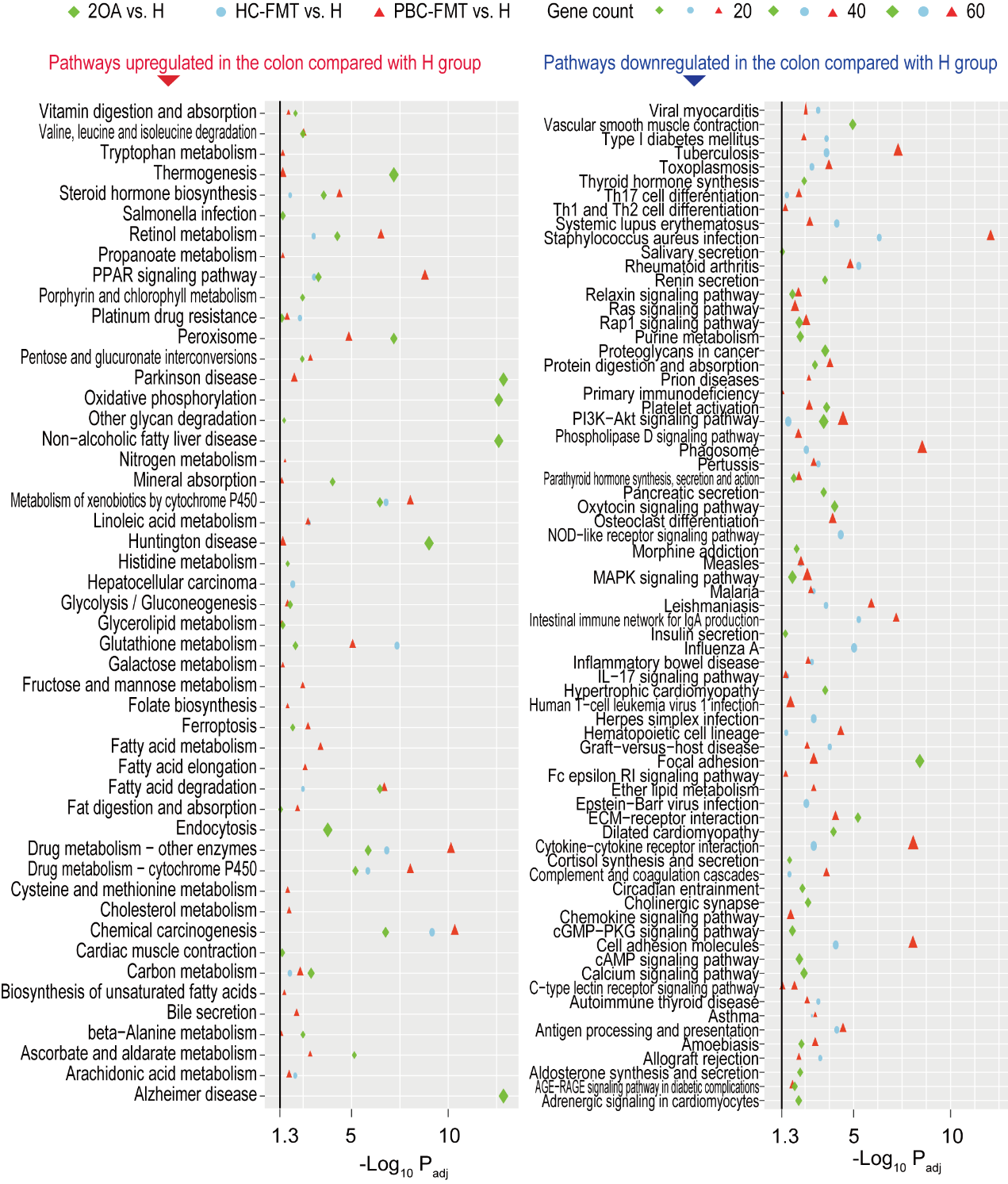
**

**Supplementary Figure 3.** **Compared with those in healthy mice, the transcript differences in the colonic genes of PBC-FMT-treated, HC-FMT-treated, and 2OA-treated mice were enriched in 86, 45 and 65 pathways, respectively.**

**
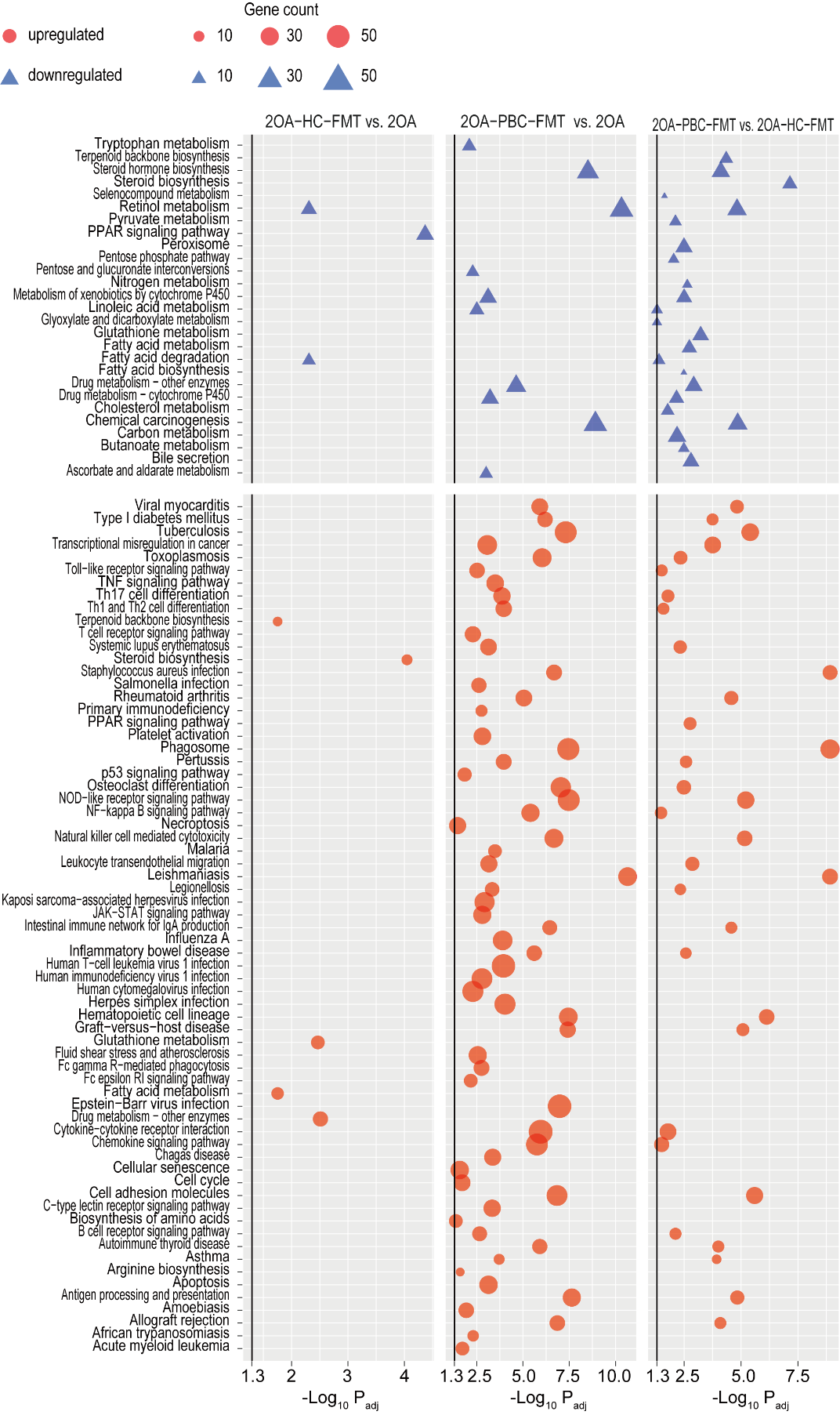
**

**Supplementary Figure 4. Pathways that were differentially enriched in the livers from 20A-HC-FMT mice compared to 20A mice, 20A-PBC-FMT mice compared to 20A mice and 20A-PBC-FMT mice compared to 20A-HC-FMT mice.**
