# Supplementary figures and images for: The fecal microbiota of patients with primary biliary cholangitis (PBC) causes PBC-like liver lesions in mice and exacerbates liver damage in a mouse model of PBC
Source: Gut Microbes. 2024 Aug 6;16(1):2383353. doi: 10.1080/19490976.2024.2383353 (PMC11305030; doi:10.1080/19490976.2024.2383353)

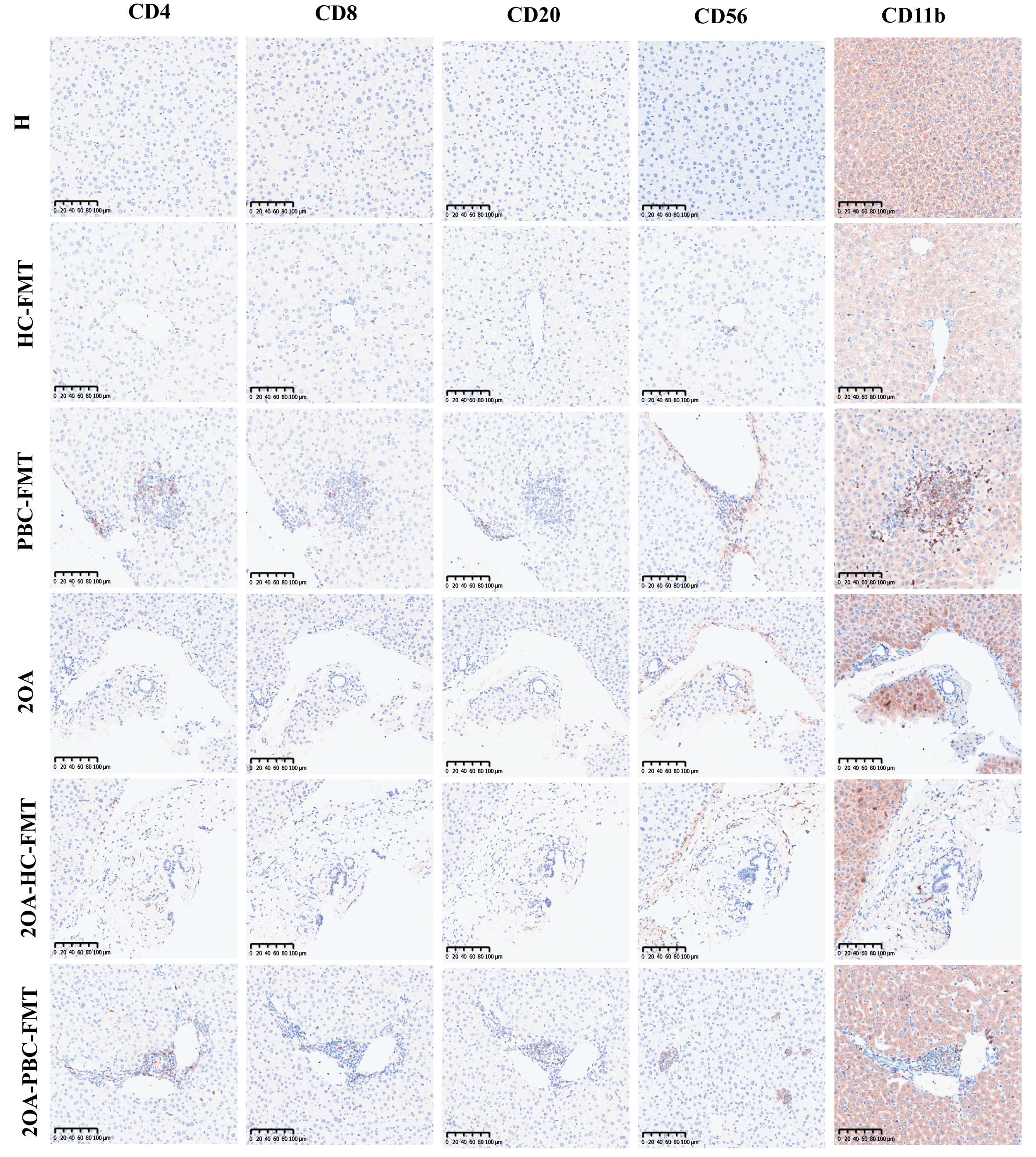

Supplement: Supplemental Material [file KGMI_A_2383353_SM2123.zip › Supplementary figuer1.tif]

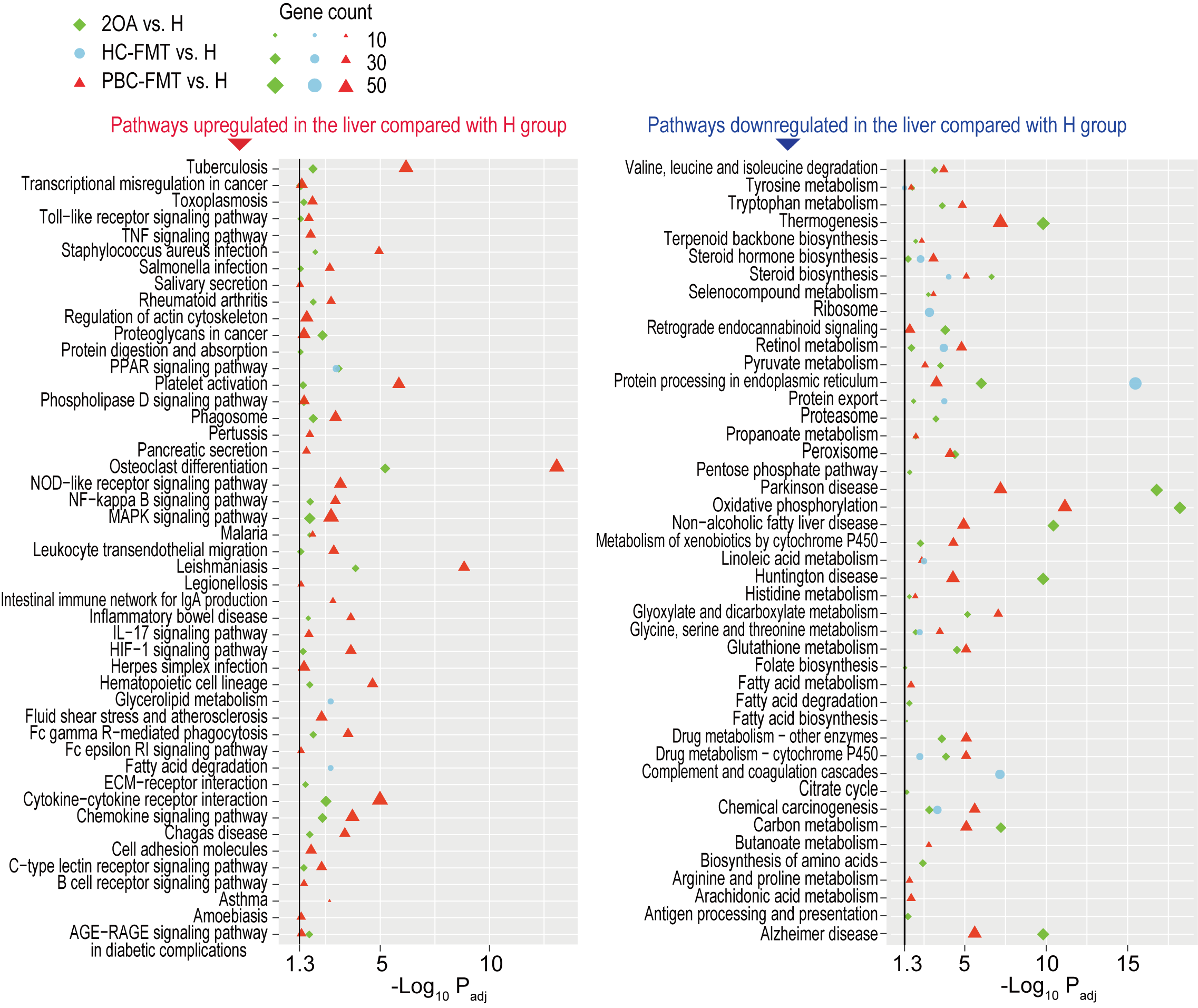

Supplement: Supplemental Material [file KGMI_A_2383353_SM2123.zip › Supplementary figuer2.tif]

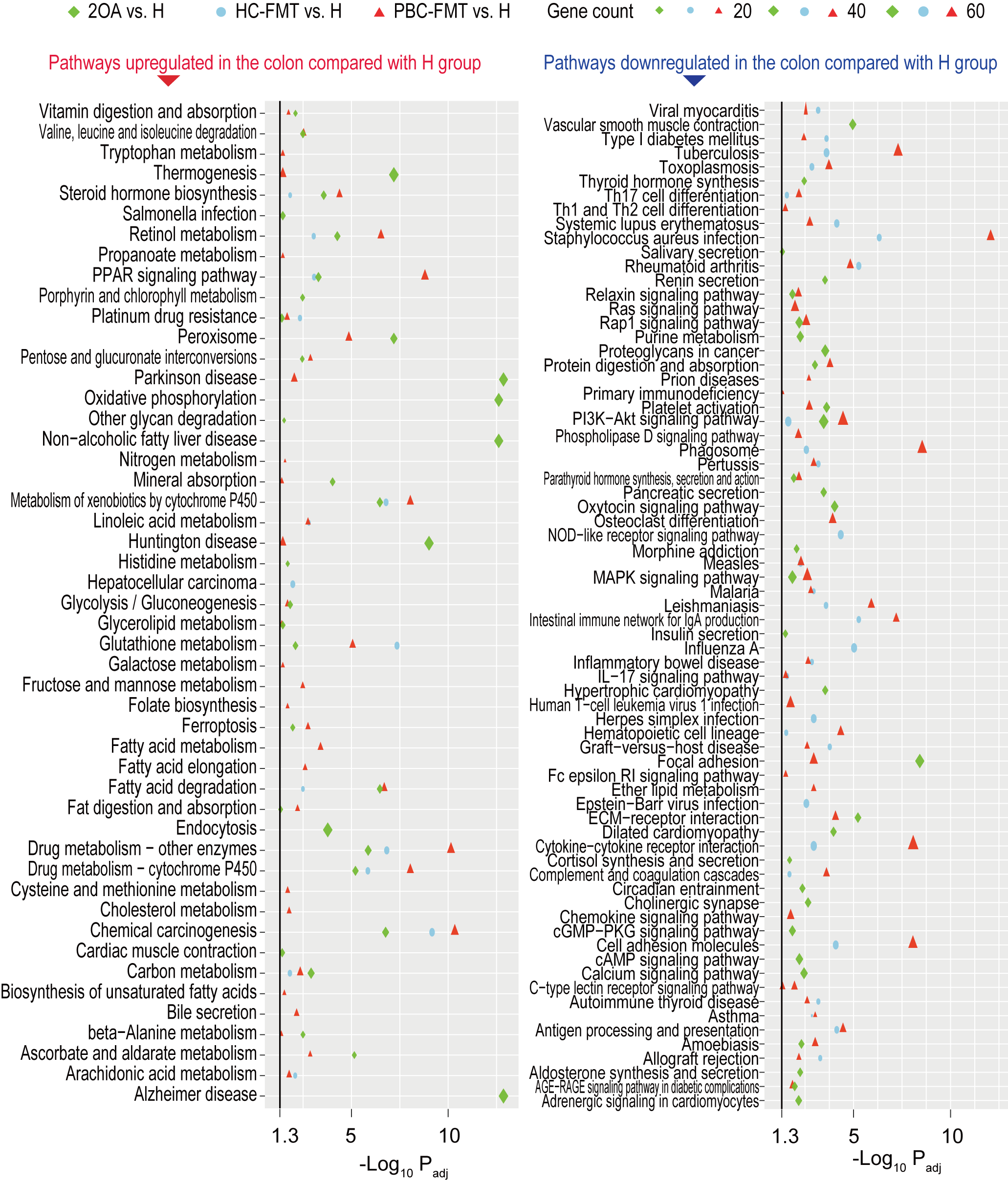

Supplement: Supplemental Material [file KGMI_A_2383353_SM2123.zip › Supplementary figuer3.tif]

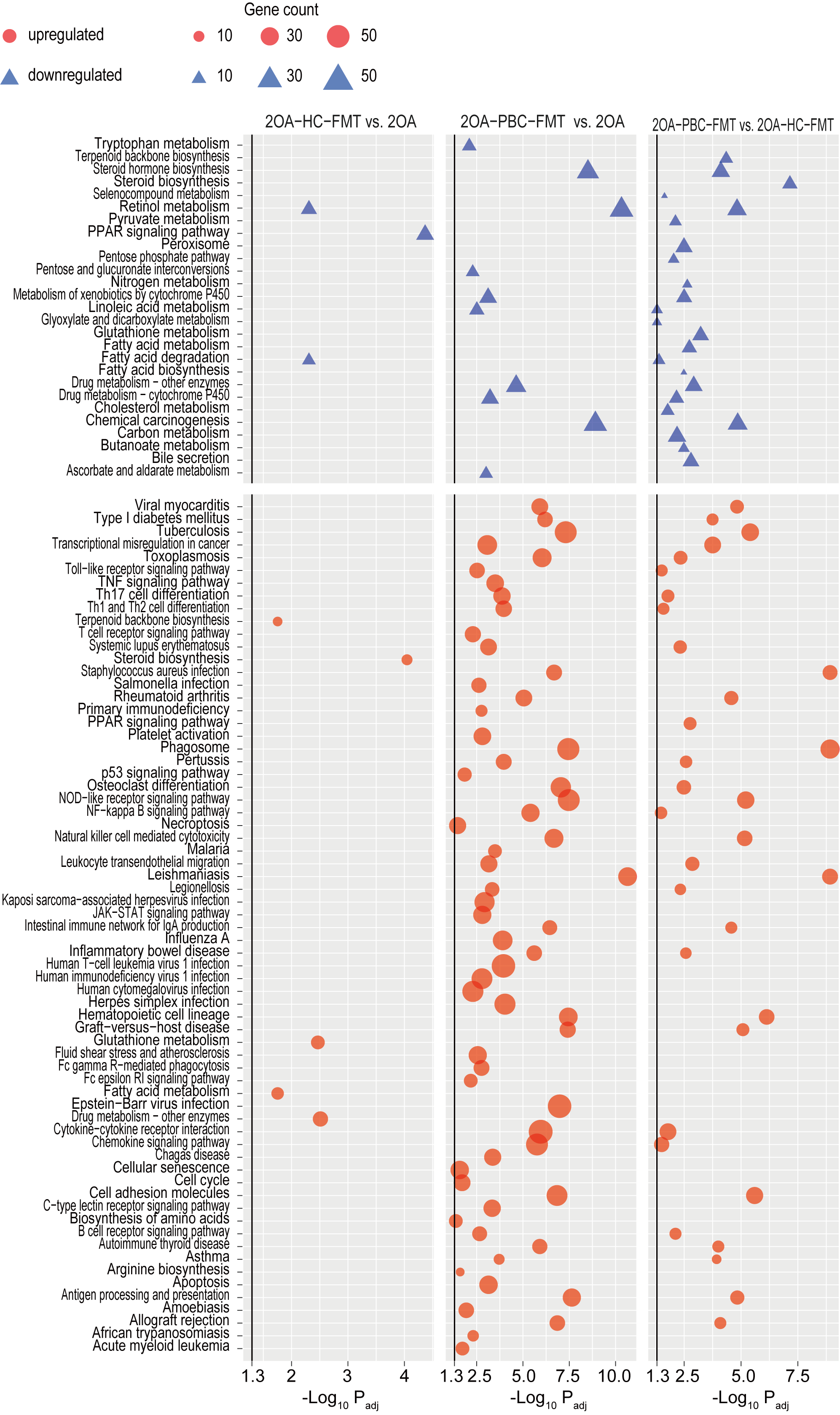

Supplement: Supplemental Material [file KGMI_A_2383353_SM2123.zip › Supplementary figuer4.tif]
